# Identification and drug metabolic characterization of four new CYP2C9 variants CYP2C9*72-*75 in the Chinese Han population

**DOI:** 10.3389/fphar.2022.1007268

**Published:** 2022-12-13

**Authors:** Fang-Ling Zhao, Qing Zhang, Shuang-Hu Wang, Yun Hong, Shan Zhou, Quan Zhou, Pei-Wu Geng, Qing-Feng Luo, Jie-Fu Yang, Hao Chen, Jian-Ping Cai, Da-Peng Dai

**Affiliations:** ^1^ The Key Laboratory of Geriatrics, Beijing Institute of Geriatrics, Institute of Geriatric Medicine, Chinese Academy of Medical Sciences, Beijing Hospital/National Center of Gerontology of National Health Commission, Beijing, China; ^2^ Peking University Fifth School of Clinical Medicine, Beijing, China; ^3^ Department of Cardiovascular, Beijing Hospital, National Center of Gerontology, Institute of Geriatric Medicine, Chinese Academy of Medical Sciences, Beijing, China; ^4^ Laboratory of Clinical Pharmacy, The Sixth Affiliated Hospital of Wenzhou Medical University, The People’s Hospital of Lishui, Lishui, China; ^5^ Department of Gastroenterology, Beijing Hospital, National Center of Gerontology, Institute of Geriatric Medicine, Chinese Academy of Medical Sciences, Beijing, China

**Keywords:** CYP2C9, drug metabolism, allelic variant, baculovirus, microsome

## Abstract

Cytochrome 2C9 (CYP2C9), one of the most important drug metabolic enzymes in the human hepatic P450 superfamily, is required for the metabolism of 15% of clinical drugs. Similar to other CYP2C family members, *CYP2C9* gene has a high genetic polymorphism which can cause significant racial and inter-individual differences in drug metabolic activity. To better understand the genetic distribution pattern of *CYP2C9* in the Chinese Han population, 931 individuals were recruited and used for the genotyping in this study. As a result, seven synonymous and 14 non-synonymous variations were identified, of which 4 missense variants were designated as new alleles CYP2C9*72, *73, *74 and *75, resulting in the amino acid substitutions of A149V, R150C, Q214H and N418T, respectively. When expressed in insect cell microsomes, all four variants exhibited comparable protein expression levels to that of the wild-type CYP2C9 enzyme. However, drug metabolic activity analysis revealed that these variants exhibited significantly decreased catalytic activities toward three CYP2C9 specific probe drugs, as compared with that of the wild-type enzyme. These data indicate that the amino acid substitution in newly designated variants can cause reduced function of the enzyme and its clinical significance still needs further investigation in the future.

## 1 Introduction

Drug administration is one of the main modalities for disease treatment in clinical trials, and the drug will experience the journey of absorption, distribution, metabolism, and excretion (ADME) in the human body. Of these processes, metabolism plays an important role in influencing drug effect, due to its contribution to the main elimination pathway of most drugs. Drug metabolism refers to the process of drug chemical structural modification *in vivo*, that directly determines the drug metabolic activity and pharmacological effects. Primarily occurring in the liver, drug metabolism is dependent on multiple hepatic microsomal enzymes, (Almazroo et al., 2017; Zhao et al., 2021), and cytochrome P450 mixed function oxidase (abbreviated as CYP450 or CYP) is the most important enzyme member because it is responsible for catalyzing 80% of clinically used drugs. (Guengerich, 2006; Subramanian et al., 2012) CYP450 enzymes, simultaneously known as monooxygenases, are a group of structurally and functionally related isozymes encoded by hemoglobin superfamily CYP genes. (Miners and Birkett, 1998) The most striking features of these CYP genes are their marked polymorphisms in different ethnic groups and geographical regions, and these genetic polymorphisms can cause a high variation in the metabolic activity of the P450 enzyme among different individuals. (Waring, 2020) To date, several public websites or organizations have been focused on the pharmacogenetics (PGx) related to drug metabolism, of which CPIC (Relling et al., 2020) and PharmVar (Sangkuhl et al., 2021) are the most important pharmacogenetic groups. Previous studies have reported that the high polymorphic status of the CYP gene is one of the crucial factors that make a great contribution to the individual differences in drug response. (Nebert and Russell, 2002; Fujikura et al., 2015) Therefore, investigating polymorphism in CYP genotypes will greatly reduce the occurrence of adverse drug reactions and help clinicians and pharmacists precisely apply diverse drugs. (Manikandan and Nagini, 2018; Qian et al., 2021)

It has been reported that the human genome includes at least 57 CYP genes, that can be divided into 18 families and 43 subfamilies based on the homologous degree of protein amino acid sequences. (Zanger and Schwab, 2013) In particular, the majority of these drugs are actually metabolized by five isoforms, CYP2C9, CYP1A2, CYP2C19, CYP2D6 and CYP3A4. (Flanagan et al., 2003) Among these five important pharmacogenetic enzymes, CYP2C9 has received extensive attention because of its marked genetic polymorphism, high expression, and large portion in drug metabolism. The *CYP2C9* gene is located on chromosome 10q24.2 with a total length of approximately 55 kb, including nine exons and eight introns. The CYP2C9 enzyme metabolizes up to 15% of clinical drugs and accounts for 20% of total cytochrome P450 protein in human liver microsomes. (Zhou et al., 2009), (Tornio and Backman, 2018) To date, more than 80 *CYP2C9* allelic variants have been identified and illustrated in the website of the Pharmacogene Variation (PharmVar) Consortium (https://www.pharmvar.org/gene/CYP2C9), in which most of them are SNPs that are located in the coding region (Gaedigk et al., 2021; Sangkuhl et al., 2021). Similar to other CYP members, *CYP2C9* gene exhibits large ethnic and individual differences in genotype and allele frequency. For example, CYP2C9*2 is the most prevalent defective allele in European with a frequency of approximately 12.7%, but it is rarely detected in the East Asian individuals (0.21%). Therefore, identifying polymorphisms across ethnic groups is crucial to understand the difference in clinical response to drugs in these populations. (DeLozier et al., 2005; Nizamuddin et al., 2021) Recently, we performed a polymorphic screening of *CYP2C9* gene in 2,127 Chinese Han individuals and found 35 allelic variants, and 21 of them were reported for the first time. (Dai et al., 2014) After that, we also identified another four defective alleles in warfarin sensitive patients. (Luo et al., 2014; Dai et al., 2015a; Dai et al., 2015b; Chen et al., 2020) These studies indicate that many other undetected *CYP2C9* allelic variants may still exist in the Chinese Han population. In this study, we performed another set of genetic screenings of the *CYP2C9* gene in Chinese individuals and detected four allelic variants, CYP2C9*72, *73, *74 and *75.

## 2 Materials and methods

### 2.1 Chemicals and materials

Materials and kits were purchased from the following sources: TIANamp Blood DNA Midi Kit (TIANGEN, Beijing, China); PrimeSTAR HS DNA polymerase and restriction enzymes (Takara Bio, Inc.; Otsu, Shiga, Japan); Spodoptera frugiperda (Sf)21 insect cells, Sf-900TM III SFM insect culture medium and Bac to-Bac Baculovirus Expression System (Invitrogen, Carlsbad, CA, United States); Mouse monoclonal anti-OR antibody (Santa Cruz Biotechnology, Dallas, Texas, United States); Rabbit polyclonal anti-CYP2C9 antibody (Abcam, Cambridge, United Kingdom); Super Signal West Pico Trial Kit (Thermo Scientific, Rockford, IL, United States); 4-Hydroxytolbutamide, 4-hydroxydiclofenac, and telmisartan (Toronto Research Chemicals, Inc.; Toronto, Ontario, Canada); Diclofenac and chlorpropamide (Tokyo Chemical Industry Co., Ltd.; Tokyo, Japan); Tolbutamide, losartan and E-3174 (Sigma-Aldrich, St. Louis, MO, United States); NADPH-regenerating system (Promega, Madison, WI, United States); High-pressure liquid chromatography-grade solvents (Fisher Scientific Co.; Fair Lawn, NJ, United States). All other chemicals and reagents were of analytical grade or the highest commercially available quality.

### 2.2 DNA extraction and genotyping

Subjects in this study (*n* = 931, 61.5% male, mean age 69) were recruited from the Physical Examination Center of Beijing Hospital and written informed consent forms were obtained from all healthy Chinese Han participants before blood sample collection. Individuals with acute or chronic diseases were exclusive and subjects with excessive drinking and smoking history were also excluded. This study was approved by the Institutional Ethics Committee of Beijing Hospital institutions. According to previously reported methods (Luo et al., 2014), genomic DNA was extracted from white blood cells using the TIANamp Blood DNA Midi Kit, and was subsequently diluted to approximately 25 ng/μL as the template for PCR amplification of the promoter or all nine exons of *CYP2C9* genes. Briefly, the experiment was carried out in a total volume of 30 μL containing 100 ng genomic DNA, 15 μL 2×Rapid Taq Master Mix (Vazyme Biotech Co. Ltd.; Nanjing, China), and 0.5 μM of each primer (Table 1). The reaction condition included an initial denaturation at 95°C for 3 min, followed by 35 cycles of 95°C for 20 s, 55°C–61°C for 15 s, and 72°C for 1–3 min, and a final extension at 72°C for 5 min. After separation by agarose gel electrophoresis, the PCR products were then sent to BioMed Biotech Company for sequencing on ABI 3730XL using the sequencing primers listed in Table 1. The acquired sequences were then aligned and compared with the reference sequence in the NCBI database with the Seqman module of DNAstar software (Version 7.1). Detected mutated sites were then compared with deposited sequences of *CYP2C9* alleles on the Nomenclature Committee website (https://www.pharmvar.org/gene/CYP2C9) for genotyping. For exons containing mutated sites not included in the PharmVar website, bidirectional sequencing was then performed for DNA sequence verification. Additionally, 2,232 bp upstream of start codon ATG was also amplified with primer pairs (5′-ACT​GAG​GCA​TTG​TGA​TTG​TGA​T-3′ and 5′- GCA​AGC​CAC​TGA​AGG​AGC​AT-3′) and sequenced with three primers (5′-AAG​GGA​AAC​AGC​ACC​AG-3′, 5′-GAG​CCT​TGA​AGA​TTC​AGT​A-3′, and 5′-GAC​TTT​GAC​CCA​CTG​ATA​CA-3′) to verify whether some other mutated sites could be detected. All the sequencing files were uploaded to PharmVar for evaluation in the new allele application process.

**TABLE 1:** Optimized primers for amplification or sequencing of human *CYP2C9* gene.

PCR primers				
Region	Forward primer (5′-3′)	Reverse primer (5′-3′)	Amplicon (bp)	Annealing temperature (°C)
Promoter + Exon1	AGA​AGC​CCT​AGT​TTC​TCA​AAC​CCT​T	TCT​ACT​CAC​AAA​ATA​CAT​GGT​TTC​A	1,419	55
Exon2+3	GCA​TCA​GTG​TTT​GAA​TAA​GCG​GA	CCC​GCT​TCA​CAT​GAG​CTA​AC	1,297	55
Exon4+5	CCA​GCT​AGG​TTG​TAA​TGG​TCA​ACT	TCA​CAA​GCA​GTC​ACA​TAA​CTA​AGC	1738	55
Exon6	TGG​GCA​AGT​TGG​TCT​ACA​GC	ACA​TGC​AAT​CCC​AGG​CCA​AT	938	61
Exon7	TGT​GCC​ATT​TTT​CTC​CTT​TTC​CAT​C	TCC​TAA​ACA​ATA​TGA​AGA​AGG​CCA​G	1,688	61
Exon8	GATTGCAGGGCACTTTA	AGGAGGAGTTCTTGGGT	639	55
Exon9	ACA​CTG​AAC​AGT​TAT​TGC​ATA​TTC​T	TGT​CCA​TTC​CAC​CCT​TTG​ACT	907	55
Sequencing primers				
Region	Sequencing primer (5′-3′)			
Promoter	AGA​AGC​CCT​AGT​TTC​TCA​AAC​CCT​T			
Exon1	AGG​CTC​CAA​CCA​AGT​ACA​GTG​AAA			
Exon2+3	TAT​TTG​AAG​CCT​GTG​TGG​CTG​AA			
Exon4	TATGAGCACGCTTTAGGG			
Exon5	TGA​TTA​TCA​TCT​GGT​TAG​AAT​TGA​T			
Exon6	AAT​CAC​CAT​TAG​TTT​GAA​ACA​GAT​TAC​AGC			
Exon7	CCT​AAG​AGT​AGC​CAA​ACC​AAT			
Exon8	GATTGCAGGGCACTTTA			
Exon9	TCT​GTC​CTT​ATC​ATT​TTG​AGA​ACC​AGC​AT			

### 2.3 Construction of dual expression vector and expression of recombinant CYP2C9 proteins in insect cells

Using previously reported methods, (Dai et al., 2013; Luo et al., 2014), cDNA of the typical defective variant CYP2C9*3 was constructed by the overlap extension PCR amplification method and used as the defective control in the functional analysis experiment. In detail, four pairs of sit-directed mutagenesis primers (5′-AGA​GGA​AG**t**CCG​CTG​CC-3’ (sense) and 5′-GGC​AGC​GG**a**CTT​CCT​CT-3’ (antisense) for *72; 5′-AGG​AAG​CC**t**GCT​GCC​TT-3’ (sense) and 5′-AAG​GCA​GC**a**GGC​TTC​CT-3’ (antisense) for *73; 5′-CCT​GGA​TCC​A**t**ATC​TGC​AAT​A-3’ (sense) and 5′-TAT​TGC​AGA​T**a**TGG​ATC​CAG​G-3’ (antisense) for *74; 5′-GAA​GGT​GGC​A**c**TTT​TAA​GAA​A-3’ (sense) and 5′-TTT​CTT​AAA​AgT​GCC​ACC​TTC-3’ (antisense) for *75. Mutated sites are illustrated as lowercase bold letters) were paired with full-length amplification primers (Forward: 5′-GCC​T*GAA​TTC*ATG​GAT​TCT​CTT​GTG​GT-3′, introducing one EcoR I site; Reverse: 5′-GAA​C*GTC​GAC*TCA​GAC​AGG​AAT​GAA​GCA-3′, introducing one Sal I site) to obtain the cDNAs of four newly detected variants. Subsequently, purified PCR products were digested with EcoRI and SalI at 37°C for 2 h. After purification, amplicons were ligated to the EcoRI/SalI double-digested pFastBac dual-OR vector, to obtain the recombinant plasmid pFastBac dual-OR-2C9. Using the Bac-to-Bac Baculovirus Expression System, these newly constructed pFastBac dual-OR-2C9 vectors were then packaged into baculoviruses for the expression of OR and 2C9 enzymes simultaneously in insect cell microsomes. Expressed CYP2C9 and OR proteins were then verified and quantified according to the methods we previously reported. (Dai et al., 2015b; Chen et al., 2020) Briefly, 0.1–0.2 pmol microsomes were separated on SDS-PAGE gels and detected using rabbit polyclonal anti-CYP2C9 antibody (AbD Serotec, Oxford, United Kingdom) or mouse monoclonal anti-OR antibody (Santa Cruz Biotechnology, Dallas, Texas, United States).

### 2.4 Enzyme kinetics analysis

To characterize the enzyme kinetic features of newly designated CYP2C9 variants, three representative CYP2C9 probe drugs, tolbutamide, diclofenac and losartan, were included in the drug metabolic activity analysis according to the methods we previously reported. (Wang et al., 2014; Dai et al., 2015b) In brief, the incubation mixture consisted of 10–20 pmol of cytochrome b5, 5–10 pmol recombinant CYP2C9 insect microsomes, 100 mM K_3_PO_4_ (pH 7.4) for tolbutamide or 100 mM Tris-HCl (pH 7.4) for diclofenac and losartan, and a series of gradient solutions of drugs (10–1,000 mM for tolbutamide, 1–100 mM for diclofenac and 0.5–25 mM for losartan). Following preincubation at 37°C for 5 min, an NADPH regeneration system (1.3 mmol/LNADP+, 3.3 mmol/L glucose-6-phosphate, 3.3 mmol/L magnesium chloride and 0.4 U/ml glucose-6-phosphate dehydrogenase) was added to start the reaction with a final reaction volume of 200 μL. The incubation proceeded at 37°C with gentle shaking for 60 min for tolbutamide or 30 min for diclofenac and losartan, and finally terminated by the addition of 200 μL acetonitrile containing 50 ng/ml diazepam as an internal standard. Samples were subsequently centrifuged at 12,000 × *g* for 5 min at 4°C and the supernatant was transferred to autosampler plastic vials for injection and detection on ACQUITY I-Class UPLC and Waters XEVO TQD MS (Milford, MA, United States). All samples were analyzed in triplicate and the whole experimental operation except for incubation was carried out on crushed ice.

The enzyme kinetic parameters Km and Vmax were calculated by GraphPad Prism (version seven; GraphPad Software, Inc. San Diego, CA, United States) with the Michaelis-Menten model and non-linear regression analysis parameters. The intrinsic clearance value was obtained by the following formula: Clint = Vmax/Km. All values are presented as the mean ± S.D. (standard deviations). IBM SPSS Statistics software (version 23.0, SPSS Inc. Chicago, IL, United States) was used for the enzymatic activity comparison between wild-type and CYP2C9 variants by one-way analysis of variance. *p* values less than 0.05 were regarded as statistically significant.

### 2.5 Homology modeling of newly designated variants

The three-dimension crystal structure of the wild-type protein CYP2C9.1 (in complex with multiple losartan, PDB ID: 5XXI) was used as a template for homology modeling on the SWISS-MODEL online website (https://swissmodel. expasy.org/) to predict the crystal structures of CYP2C9.72 and CYP2C9.74. PyMOL software (Version 2.7, Schrodinger, LLC) was then used for the visualization and alignment of crystal structures of CYP2C9.1 with predicted models.

## 3 Result

### 3.1 CYP2C9 genotyping in the Chinese han population

**FIGURE 1:**
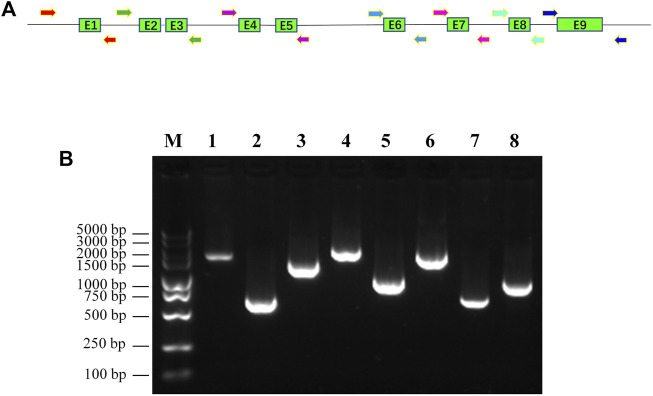
Amplification of promoter region and exons of human *CYP2C9* gene with newly designed PCR primers. **(A)**: Diagram of newly designed primers and genomic structure of human *CYP2C9* gene. Shaded boxes represent the exons and lines indicate the promoter region and introns. Arrowheads indicate the primers that used for the PCR amplification. **(B)**: Gel picture of PCR amplicons of *CYP2C9* promoter and exons. M: DNA molecular standard; 1: promoter region; 2: exon1; 3: exon2+exon3; 4: exon4+exon5; 5: exon6; 6: exon7; 7: exon8; 8: exon9.

**FIGURE 2:**
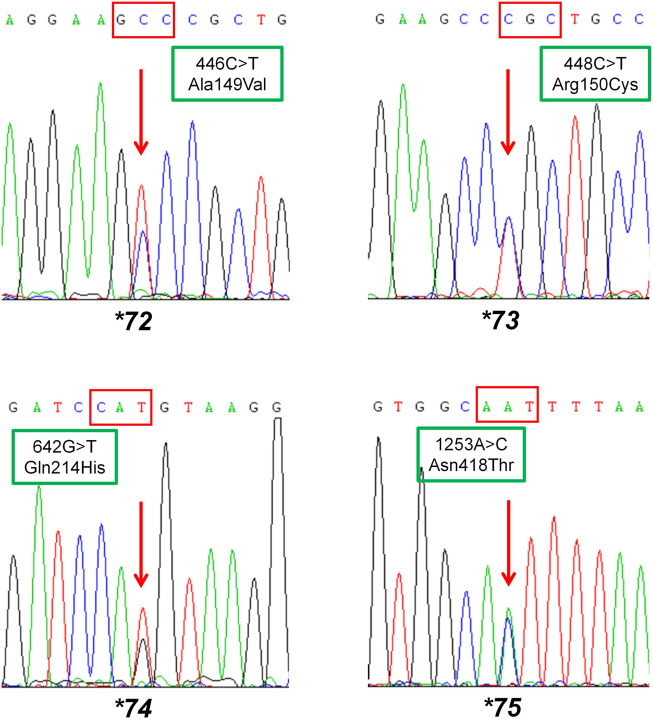
Sequencing results of the carriers with newly designated alleles CYP2C9*72, CYP2C9*73, CYP2C9*74 and CYP2C9*75. The red arrow indicates mutated sites in allelic variants CYP2C9*72-*75. Rectangular boxes show the cDNA mutations and the amino acid substitutions at codon 149, 150, 214 and 418, respectively.

To fit the standard for new CYP variant submission on the Pharmacogene Variation Consortium (PharmVar), we redesigned and optimized the primers for PCR amplification and sequencing (Table 1; Figure 1A). As illustrated in Figure 1B, all the target regions of *CYP2C9* could be successfully amplified and sequenced with high quality. After sequencing 931 healthy Chinese Han individuals, a total of seven synonymous polymorphic sites and 10 known *CYP2C9* alleles were detected in this study (Table 2; Table 3). In addition, four non-synonymous SNVs (single nucleotide variants) were also identified and designated as CYP2C9*72, CYP2C9*73, CYP2C9*74 and CYP2C9*75 by the Pharmacogene Variation Consortium (PharmVar, https://www.pharmvar.org/gene/CYP2C9). These newly designated allelic variants of *CYP2C9* are located in exon 3 (for *72 and *73), exon 4 (for *74) or exon 8 (for *75) of the gene and carriers with these alleles are all heterozygotes. As shown in Figure 2, CYP2C9*72 contains a SNV of 446C>T and is estimated to make an amino acid substitution of alanine by valine at position 149 of the CYP2C9 protein (A149V). CYP2C9*73 has another type of SNV 448C>T that can lead to the replacement of arginine with cysteine at position 150 of the protein (R150C). CYP2C9*73 processes the nucleotide alternation from G to T at position 642 of cDNA, resulting in the amino acid substitution of glutamine by histidine at position 214 (Q214H). CYP2C9*75 has an A to C variation at position 1,253 of cDNA that contributes to the replacement of asparagine by threonine at position 418 of CYP2C9 protein (N418T). No other mutated sites could be detected within the promoter region and splicing sites of the *CYP2C9* gene in carriers.

**TABLE 2:** Synonymous and missense mutations of *CYP2C9* in the Chinese Han population.

gDNA position	Region	Effect on protein	Allele	rsID	n	Allelic frequency (%)	SIFT^a^	Polyphen-2^b^
108C>G	Exon1	L36L	/	rs1311013151	1	0.05		
3235G>A	Exon2	V76V	/	rs17847036	8	0.43		
3276T>C	Exon2	L90P	*13	rs72558187	1	0.05	0.092	0.9
3549G>A	Exon3	R124Q	*42	rs12414460	1	0.05	0.006	1
3573G>A	Exon3	R132Q	*33	rs200183364	1	0.05	0.003	0.973
3608C>T	Exon3	R144C	*2	rs1799853	3	0.16	0.028	1
3623G>A	Exon3	A149T	*46	rs754487195	1	0.05	0.034	0.944
3624C>T	Exon3	A149V	*72	rs1289704600	1	0.05	0.032	0.996
3626C>T	Exon3	R150C	*73	rs17847037	1	0.05	0.079	0.084
3627G>T	Exon3	R150L	*27	rs7900194	1	0.05	0.318	0.003
8757G>T	Exon4	Q214H	*74	/	1	0.05	0	0.999
9216T>C	Exon4	L201L	/	rs1429669733	2	0.11		
10464G>A	Exon5	P227P	/	rs772651628	1	0.05		
10491C>T	Exon5	N236N	/	rs765176937	2	0.11		
33437C>A	Exon6	P279T	*29	rs182132442	3	0.16	0.158	0
42614A>C	Exon7	I359L	*3	rs1057910	98	5.26	0.013	0.002
42676T>C	Exon7	Y379Y	/	rs141283168	24	1.29		
47454A>C	Exon8	N418T	*75	rs1254213342	1	0.05	0.227	0.088
50173A>T	Exon9	I434F	*59	/	1	0.05	0.005	0.969
50273T>C	Exon9	L467P	*60	rs767284820	2	0.11	0	0.994
50298A>T	Exon9	G475G	/	rs1057911	8	0.43		

Slash (/) represents not applicable.

a SIFT, value < 0.05 is regarded as ‘Deleterious’ and ≥0.05 is regarded as ‘Tolerated’.

b Polyphe-2, value ≥ 0.05 is predicted as ‘Probably damaging’, 0.447 ≤ value ≤0.909 is predicted as ‘Possibly damaging, and ≤0.446 is predicted as ‘Benign’.

**TABLE 3:** Genotype frequencies of *CYP2C9* allelic variants in Han Chinese populations population.

Genotype	n	Frequency (%)
*1/*1	818	87.86
*1/*2	3	0.32
*1/*3	92	9.88
*3/*3	3	0.32
*1/*13	1	0.11
*1/*27	1	0.11
*1/*29	3	0.32
*1/*33	1	0.11
*1/*42	1	0.11
*1/*46	1	0.11
*1/*59	1	0.11
*1/*60	2	0.21
*1/*72	1	0.11
*1/*73	1	0.11
*1/*74	1	0.11
*1/*75	1	0.11

### 3.2 Expression and functional characterization of the CYP2C9 variants

**FIGURE 3:**
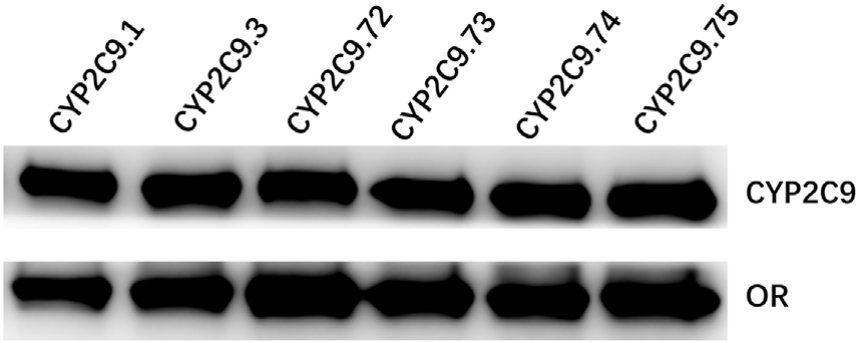
Western blotting analysis of expressed wild-type and five CYP2C9 variants in insect cell microsomes. CYP2C9.1, CYP2C9.3, CYP2C9.72, CYP2C9.73, CYP2C9.74 or CYP2C9.75 were highly co-expressed with OR in insect cell microsomes.

**FIGURE 4:**
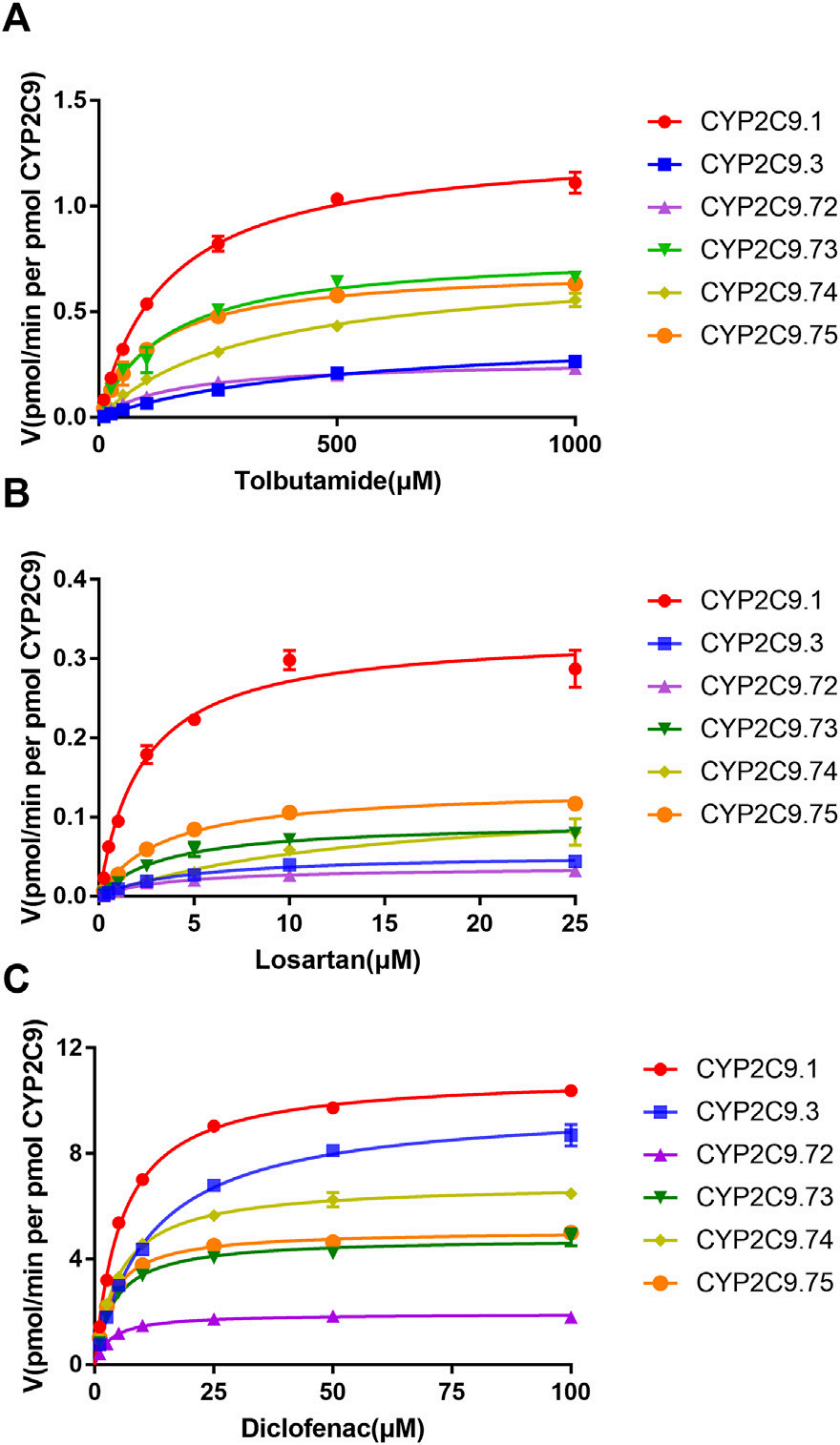
Michaelis–Menten curves for the hydroxylation of three CYP2C9 probe substrates tolbutamide **(A)**, losartan **(B)** and diclofenac **(C)** by the recombinant CYP2C9 variants. Each point represents the mean ± standard deviation of at least three independent experiments.

To better investigate the impacts of these newly found CYP2C9 variants on protein expression and drug metabolic activity, the baculovirus based insect cell expression system was used for CYP2C9 expression and microsome preparation. (Dai et al., 2015a; Liu et al., 2021) Immunoblot results revealed that both OR and 2C9 enzymes were successfully co-expressed in insect microsomes and all newly detected variants had similar protein expression levels to that of the wild-type CYP2C9 enzyme (Figure 3). Typically, tolbutamide (TOL), diclofenac (DIC) and losartan (LOS) are three probe drugs for CYP2C9 because only CYP2C9 mediates their metabolism in human liver microsomes (Waring, 2020). In this study, the drug metabolic activity of newly designated variants was investigated with these drugs as substrates. As a result, CYP2C9.72 (Relative Clearance of TOL, LOS, DIC is only 17.90%, 5.54% and 24.73% of wild-type CYP2C9.1, respectively, with *p* < 0.05) and CYP2C9.74 (Relative Clearance of TOL, LOS, DIC is 24.55%, 5.28%, and 55.5% of CYP2C9.1, respectively, with *p* < 0.05) exhibited comparable or even lower catalytic activities to that of the typical defective variant CYP2C9.3, and the other two variants CYP2C9.73 (Relative Clearance of TOL, LOS, DIC is 60.50%,15.53% and 48.88% of CYP2C9.1, respectively, with *p* < 0.05) and CYP2C9.75 (Relative Clearance of TOL, LOS, DIC is 64.85%, 23.64% and 61.89% of CYP2C9.1, respectively, with *p* < 0.05) also showed significantly decreased activities relative to that of wild-type (Figure 4 and Table 4). These data indicated that the amino acid substitutions in variants A149V and Q214H had greater impacts on enzyme’s drug metabolic activity than the amino acid replacements in variants R150C and N418T.

**TABLE 4:** Enzyme kinetic values of recombinant wild-type and CYP2C9 variants.

Substrate	Allelic variants	Vmax (pmol/min/pmol P450)	Km (μM)	Clearance (Vmax/Km)	Relative clearance (/CYP2C9.1)
Tolbutamide	CYP2C9.1	1.30 ± 0.065	143.60 ± 15.027	0.0091 ± 0.00049	100.00%
	CYP2C9.3	0.40 ± 0.038*	501.43 ± 65.61*	0.00081 ± 0.000051*	8.93%*
	CYP2C9.72	0.27 ± 0.011*	168.70 ± 22.13	0.0016 ± 0.00015*	17.90%*
	CYP2C9.73	0.79 ± 0.010*	144.30 ± 14.65	0.0055 ± 0.00051*	60.50%
	CYP2C9.74	0.73 ± 0.064*	334.13 ± 57.95	0.0022 ± 0.0018*	24.55%*
	CYP2C9.75	0.71 ± 0.020*	122.50 ± 20.42	0.0059 ± 0.00076*	64.85%
Losartan	CYP2C9.1	0.37 ± 0.026	2.16 ± 0.39	0.17 ± 0.020	100.00%
	CYP2C9.3	0.058 ± 0.0024*	4.42 ± 0.39*	0.013 ± 0.00064*	7.61%*
	CYP2C9.72	0.038 ± 0.0012*	3.94 ± 0.38*	0.0010 ± 0.00066*	5.54%*
	CYP2C9.73	0.094 ± 0.0034*	3.55 ± 0.50	0.027 ± 0.0038*	15.53%*
	CYP2C9.74	0.14 ± 0.046*	15.53 ± 7.00	0.0091 ± 0.0010*	5.28%*
	CYP2C9.75	0.14 ± 0.0046*	3.35 ± 0.035	0.041 ± 0.0013*	23.64%*
Diclofenac	CYP2C9.1	10.55 ± 0.051	4.43 ± 0.21	2.38 ± 0.10	100.00%
	CYP2C9.3	9.69 ± 0.14*	11.65 ± 0.57*	0.83 ± 0.029*	34.95%*
	CYP2C9.72	1.94 ± 0.028*	3.29 ± 0.11*	0.59 ± 0.014*	24.73%*
	CYP2C9.73	5.40 ± 0.60*	5.59 ± 1.70	1.00 ± 0.17*	48.88%*
	CYP2C9.74	6.86 ± 0.11*	5.19 ± 0.29	1.32 ± 0.055*	55.5%*
	CYP2C9.75	4.91 ± 0.26*	3.16 ± 0.42	1.57 ± 0.13*	61.89%

Data are presented as the mean ± S.D., of three different expression experiments. *p < 0.05 vs wild-type. All data were analyzed by one-way analysis of variance.

### 3.3 Homology modeling of variants

**FIGURE 5:**
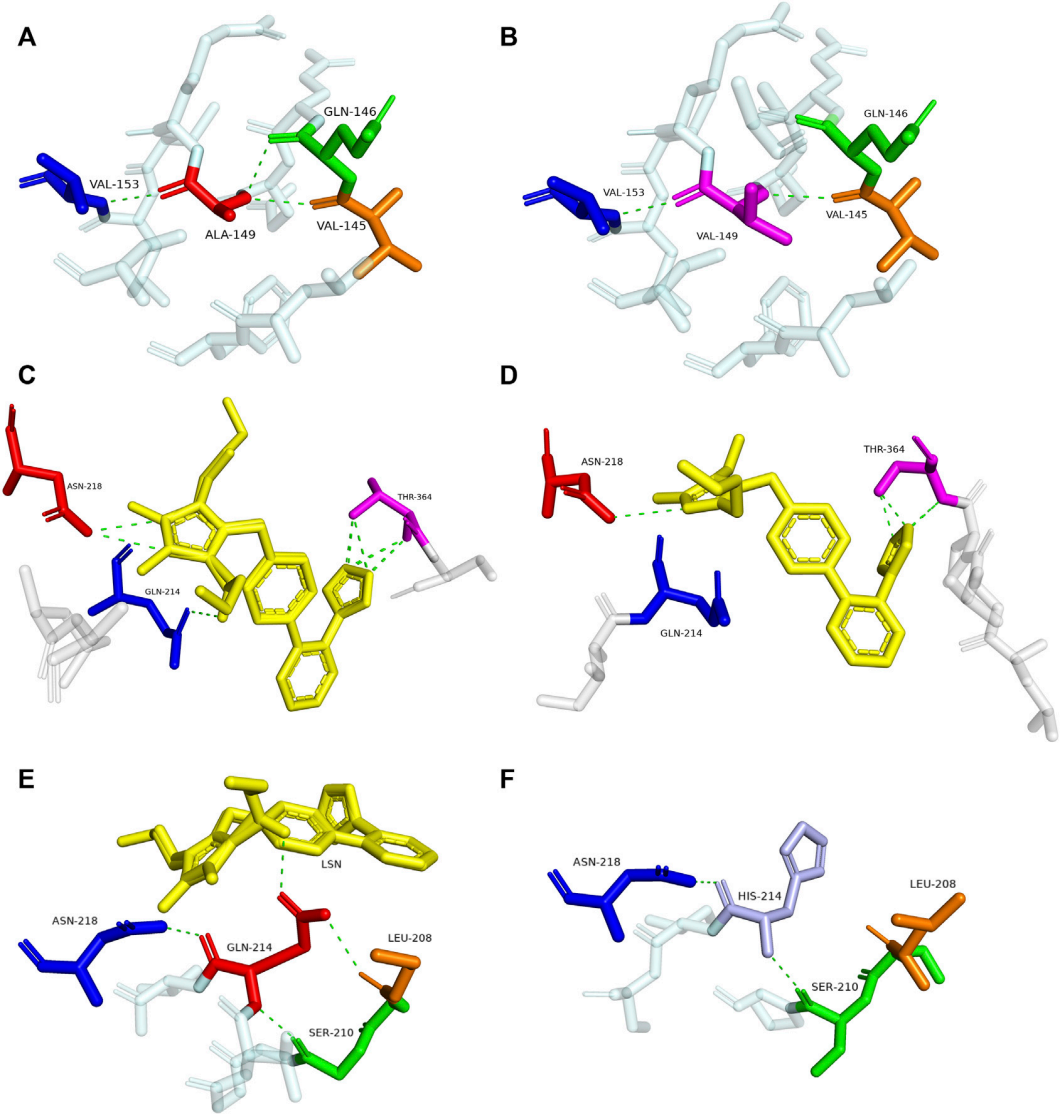
Magnified view of the crystal structure of amino acids within 5 Å around the mutated site of CYP2C9 variants and display of residues interacting with losartan. The hydrogen bonds are illustrated with green dashed lines. Replaced residues of Ala149 in CYP2C9.72, Gln214 in CYP2C9.74 and their interacted residues are shown in **(B,F)**. Corresponding structures of CYP2C9.1 are also presented in **(A,E)** to illustrate the main difference between wild-type enzyme and newly detected variants. Crystal structures of losartan-interacting amino acid residues in wild-type CYP2C9.1 and variant CYP2C9.72 are displayed in **(C,D)**, respectively. The ligand losartan is shown with yellow color and the hydrogen bonds are shown with green dashed lines.

To explore the mechanism for the decreased drug metabolic activity of newly designated variants, homology modeling was performed to predict the impacts of amino acid substitutions on the interactions between the substrate and critical residues of the CYP2C9 enzyme. Relative to that of the wild-type enzyme, all variants exhibited more than 75% reduction in the clearance rate for losartan (Figure 4; Table 4). Thus, the crystal structure of losartan-combined wild-type CYP2C9 (PDB ID 5XXI) was selected as the template for homology modeling in this study. CYP2C9.72 and CYP2C9.74 were chosen as homology modeling objects to compare 3D structures with the wild-type CYP2C9, due to their extremely lower metabolic activities relative to that of the other two variants. Focusing on the structure and amino acids within 5 Å around the mutated sites, alanine at position 149 of wild-type CYP2C9 could form three hydrogen bonds with surrounding residues Q146, V145 and V153 (Figure 5A). However, the hydrogen bond between residues Q146 and V149 in CYP2C9.72 was lost (Figure 5B), which might impact the structural stability of the D helix. (Cojocaru et al., 2007) In addition, amino acid substitution in residue 149 also leads to the missing or reduced numbers of hydrogen bonds between losartan and surrounding residues Thr364, Asn218, and Gln214 (Figures 5C,D). We speculated that missing of these hydrogen bonds in variants CYP2C9.72 might affect the stability of CYP2C9 protein and weaken the interactions between the ligand losartan and surrounding residues, which in turn leads to the decrease in the metabolic activity of CYP2C9 enzyme. It has been reported that Glutamine at position 214 is one of the key amino acids of CYP2C9 enzyme because it helps to form the F-G loop and participates in the composition of the active site channel (Williams et al., 2003) Our homology modeling results showed that the amino acid replacement of glutamine by histidine at position 214 results in the missing of hydrogen bond between His214 and Leu208, and causes the blocking of losartan into the active pocket of CYP2C9 enzyme (Figures 5E, F). We suspect that the blockage of losartan entrance might responsible for the significantly decreased metabolic activity of variant CYP2C9.74.

## 4 Discussion

After being expressed in insect microsomes, all newly detected allelic variants in this study exhibited similar protein expression levels to wild-type but showed significantly reduced drug metabolic activities toward three probe drugs, although the extent of decline in metabolic activity for each variant varied for different substrates (Figure 3; Figure 4). Interestingly, we previously reported another allele CYP2C9*46 that can produce a variant with an amino acid substitution of alanine with threonine at position 149 (A149T). Subsequent *in vitro* functional evaluation revealed that variant CYP2C9.46 was also a poor metabolism mutation type but showed a significantly lower protein expression level than the wild-type. (Dai et al., 2014) In addition, two allelic variants, CYP2C9*8 (R150H) and CYP2C9*27 (R150L), were also reported to have amino acid substitutions at position arginine 150 of the CYP2C9 protein (Rettie et al., 1994; Blaisdell et al., 2004; Maekawa et al., 2006) Studies on their drug metabolic activities showed that compared to the wild-type CYP2C9.1, variant CYP2C9.8 exhibited a higher clearance rate for tolbutamide *in vitro* but showed little difference in losartan metabolism *in vivo*. (Allabi et al., 2004; Blaisdell et al., 2004) For variant CYP2C9.27, it exhibited kinetic parameters against diclofenac similar to those of the wild-type enzyme when expressed in COS-1 cells. (Maekawa et al., 2006) Additionally, another type of amino acid substitution at position 214 (Q214L for CYP2C9*28) has been previously reported in the Japanese population. Similar to our data for Q214H, functional analysis results showed that this allelic variant of CYP2C9 exhibited two-fold higher Km values and three-fold lower Vmax values than wild-type and carriers with CYP2C9*28 could be regarded as Poor metabolizer. (Maekawa et al., 2006; Maekawa et al., 2009) These data indicated that amino acid substitutions at different positions had different impacts on the catalytic activities toward different CYP2C9 probe substrates.

To better understand the mechanism behind the reduction in catalytic activity of new CYP2C9 variants toward losartan, homology modeling of CYP2C9.72 and CYP2C9.74 was performed through the SWISSMODEL website. Crystal structure investigation revealed that Ala149 is far from the active center of heme and located in the D-helix center. However, the amino acid substitution of Ala by Val at position 149 can lead to a decrease in hydrogen bonds between mutated sites and surrounding amino acids (Figure 5B), which may result in the instability of the D-helix center. (Williams et al., 2003) In addition, the homology modeling results in this study also indicated that the amino acid substitution of A149V could weaken the interactions between losartan and the surrounding amino acid residues within 5 Å Q214, N218 and T364 (Figure 5D). Previous studies have shown that residues Q214 and N218 are involved in the formation of F0 and G0 in the F-G ring, which is the most important structure on top of the active CYP channel (Williams et al., 2003; Wester et al., 2004). When combined with losartan, the polar residues Q214 and N218 in CYP2C9.1 can interact with the imidazole ring of losartan by hydrogen bonds, with the hydroxyl portion of the Q214-linked imidazole ring acting a potential activation site for losartan metabolism. (Maekawa et al., 2017) In addition, the tetrazole of losartan could contact the polar side chain of the T364 residue in the wild-type enzyme to form eight hydrogen bonds (Figure 5C). These interactions suggest that residues Q214, N218 and T364 play vital roles in the metabolism of losartan. Previous reports have proven that amino acid changes in Q214 could have a great impact on the enzyme’s drug metabolic activity. For example, variant CYP2C9.28 (containing Q214L amino acid substitution) exhibited an 87% and 73% reduction in the metabolism of losartan and diclofenac, respectively, compared with that of the wild-type enzyme. (Maekawa et al., 2006; Maekawa et al., 2009) Our homology modeling results indicated that the amino acid replacement of Gln214 with histidine in variant CYP2C9.74 may cause the missing of interactions between losartan ligand and enzyme (Figure 5F). We believed that the interaction lost between losartan and enzyme in variant CYP2C9.74 and the hydrogen bonds lost between losartan and key residues Q214, N218 or T364 in CYP2C9.72 are likely to be one of the main reasons for their decreased drug metabolic activity.

In addition to four newly designated allelic variants, CYP2C9*72-*74, ten previously reported alleles *2, *3, *13, *27, *29, *33, *42, *46, *59 and *60 were also detected in this study (Table 2). Similar to our previously reported results (Dai et al., 2014), CYP2C9*3 was the most prevalent *CYP2C9* allelic variant in the Chinese Han population with an allele frequency of 5.26% and a genotype frequency of 9.88% for *1/*3 (Table 3). Alleles *13, *42, *46, *59 and *60 are all CYP2C9 allelic variants that were first reported in the Han Chinese population. Functional evaluation revealed that these five variants exhibited significantly decreased catalytic activities toward probe drugs *in vitro* (Dai et al., 2013; Dai et al., 2014; Dai et al., 2015b). CYP2C9*27, *29 and *33 are rare alleles that were first detected in the Japanese population, in which *29 and *33 showed significantly reduced metabolic activity against tolbutamide while *27 exhibited similar activity to that of wild-type enzyme. (Maekawa et al., 2006) These data and our current results indicate that many rare *CYP2C9* allelic variants exist in East Asian populations, represented by Chinese and Japanese individuals, and most of them can lead to a reduction in the drug metabolic activity of enzymes.

In this study, only one individual was discovered to carry CYP2C9*72, *73, *74 or *75 indicating that they all belong to the rare *CYP2C9* alleles in the Chinese Han population, and genetic screening in a large sample size is still needed to further elucidate their real allele frequencies in the Chinese population. With the development of sequencing techniques, many rare *CYP2C9* variants have been identified by large-scale genome sequencing projects, such as the 100,000 Genomes Project (Marx, 2015) and the RIGHT protocol study (Bielinski et al., 2014). In the population database gnomAD (version 2.1.1) (Karczewski et al., 2020), 409 missense variants have been deposited and 217 of them are singletons (http://gnomad-sg.org/). A149V (*72), R150C (*73) and Q214H (*74) are all included in the gnomAD database, in which A149V is only detected in African/African-American with a frequency of 0.0000106; R150C can be found in South Asian, European, African/African-American and other populations with a frequency from 0.00005 to 0.000392; Q214H is detected in only European people with a frequency of 0.0000088. These data indicated that these *CYP2C9* allelic variants are rarely detected in East Asian population, although we first reported their appearance in the Chinese Han population. To date, the vast majority of detected variants of *CYP2C9* are still unannotated. To address this challenge, the deep mutational scanning (MDS) method was developed recently for the evaluation of the enzyme activity of missense variants on a high throughput scale (Kinney and McCandlish, 2019). Zhang et al. developed a “landing pad” DMS system to determine the protein expression level of 109 missense variants of CYP2C9 in HEK 293T cells (Zhang et al., 2020). In that study, R150C was included and exhibited a similar protein expression level to that of the wild-type, which is in agreement with our current study. However, their system cannot analyze the drug metabolic activity for each variant. Recently, Amorosi et al. constructed a CYP2C9 library with more than 6,000 missense variants in both yeast and 293T cells by saturation mutagenesis and developed a multiplexed sequencing-based method, click-seq, for the multiplexed assay of CYP2C9 enzymatic activity (Amorosi et al., 2021). The advantage of this system is that it can evaluate both protein expression capacity and metabolic activity for thousands of constructed variants. In their yeast-cell-based system, both R150C and N418T were classified as “decreased variant”, while the other two variants A149V and Q214H were classified as “possibly non-sense-like” and “wt-like”, respectively. However, their system could not provide the detailed kinetic characterization for some specific drug substrates. In the current study, we reported four CYP2C9 allelic variants A149V, R150C, Q214H and N418T in the Chinese Han population and presented their kinetic characteristics for three CYP2C9 probe drugs tolbutamide, diclofenac and losartan. Our data indicated that all variants exhibited significantly reduced drug metabolic activity than the wild-type. SIFT (Sorting Intolerant From Tolerant) and PolyPhen-2 are two widely used tools for the computational predictions of the impact of protein sequence variants. (Ng and Henikoff, 2003; Adzhubei et al., 2010) Consistent to our data, CYP2C9*72 and *74 are predicted as Deleterious or Probably Damaging by SIFT or PolyPhen-2, respectively. However, CYP2C9*73 and *75 are predicted to have no impacts on enzyme’s activity by both SIFT and PolyPhen-2 tools (Table 2), indicating that computational prediction may cause misinterpretation for some specific CYP variants. Recently, reported a well-validated workflow integrating the next-generation sequencing, *in silico* analysis and *in vitro* validation assays together, in order to identify the rare and novel pharmacogenomic (PGx) variants and evaluate their functional effects on enzyme’s activity. Siamoglou et al. (2022) However, that system is only focused on CYP2C19 and CYP2D6 and it still needs the incorporation of *in vitro* assay data into program training process to enrich the prediction potential and improve its accuracy. Expanding of this type of computational prediction to CYP2C9 and coupling it with the drug metabolic activity evaluation data will benefit the proper interpretation of rare CYP2C9 variants and assist its application for personalized medicine in clinical practice.

In conclusion, CYP2C9*3 is the most common *CYP2C9* allelic variant in the Chinese Han population, although other rare variants are also detected. Additionally, four allelic variants (CYP2C9*72, *73, *74 and *75) were discovered and functional evaluation results indicated that these variants exhibited reduced metabolic activity toward CYP2C9 probe drugs. This study enriches the knowledge of the pharmacogenomics of *CYP2C9* and provides important theoretical guidance for the establishment of individualized dosing guidelines for the Chinese Han population, although further clinical investigation is still needed in the future.

## Data Availability

The datasets presented in this study can be found in online repositories. The names of the repository/repositories and accession number(s) can be found below: https://www.ncbi.nlm.nih.gov/snp/, rs1289704600, https://www.ncbi.nlm.nih.gov/snp/, rs17847037.
